# A Study on Distinguishing ChatGPT-Generated and Human-Written Orthopaedic Abstracts by Reviewers: Decoding the Discrepancies

**DOI:** 10.7759/cureus.49166

**Published:** 2023-11-21

**Authors:** Konstantinos G Makiev, Maria Asimakidou, Ioannis S Vasios, Anthimos Keskinis, Georgios Petkidis, Konstantinos Tilkeridis, Athanasios Ververidis, Efthymios Iliopoulos

**Affiliations:** 1 Department of Orthopaedics, University General Hospital of Alexandroupolis, Democritus University of Thrace, Alexandroupoli, GRC; 2 School of Medicine, University General Hospital of Alexandroupolis, Democritus University of Thrace, Alexandroupoli, GRC

**Keywords:** orthopaedic abstracts, identification, reviewers, ai detector, artificial intelligence, chatgpt

## Abstract

Background: ChatGPT (OpenAI Incorporated, Mission District, San Francisco, United States) is an artificial intelligence (AI)-based language model that generates human-resembling texts. This AI-generated literary work is comprehensible and contextually relevant and it is really difficult to differentiate from human-written content. ChatGPT has risen in popularity lately and is widely utilized in scholarly manuscript drafting. The aim of this study is to identify if 1) human reviewers can differentiate between AI-generated and human-written abstracts and 2) AI detectors are currently reliable in detecting AI-generated abstracts.

Methods: Seven blinded reviewers were asked to read 21 abstracts and differentiate which were AI-generated and which were human-written. The first group consisted of three orthopaedic residents with limited research experience (OR). The second group included three orthopaedic professors with extensive research experience (OP). The seventh reviewer was a non-orthopaedic doctor and acted as a control in terms of expertise. All abstracts were scanned by a plagiarism detector program. The performance of detecting AI-generated abstracts of two different AI detectors was also analyzed. A structured interview was conducted at the end of the survey in order to evaluate the decision-making process utilized by each reviewer.

Results: The OR group managed to identify correctly 34.9% of the abstracts’ authorship and the OP group 31.7%. The non-orthopaedic control identified correctly 76.2%. All AI-generated abstracts were 100% unique (0% plagiarism). The first AI detector managed to identify correctly only 9/21 (42.9%) of the abstracts’ authors, whereas the second AI detector identified 14/21 (66.6%).

Conclusion: Inability to correctly identify AI-generated context poses a significant scientific risk as “false” abstracts can end up in scientific conferences or publications. Neither expertise nor research background was shown to have any meaningful impact on the predictive outcome. Focus on statistical data presentation may help the differentiation process. Further research is warranted in order to highlight which elements could help reveal an AI-generated abstract.

## Introduction

ChatGPT (OpenAI Incorporated, Mission District, San Francisco, United States) is a large language model (LLM) developed by OpenAI. It is essentially a chatbot, which is based on artificial intelligence (AI) technology with free public access. ChatGPT is a member of a bigger LLM family called Generative Pre-trained Transformers (GPT) that extracts information from an abundant data store and is specialized to generate human-resembling responses based on users’ prompts or inputs [1].

This AI application has recently attracted attention, especially within the scientific community, and raised a lot of scientific debate. The reason behind its popularity lies in the fact that it can compose quickly and effortlessly a comprehensible and contextually relevant literary work on a variety of subjects. This AI-generated text can even be utilised for the population of scientific or research content [2]. ChatGPT has interestingly been included among authors in several published articles and is even catalogued within Scopus’ database with its own registered ORCID ID [3,4]. It has also been reported for its outstanding performance in response to a challenge, delivering high-rated college essays and managing to impress when taking board-like examinations for radiology and orthopaedic surgery [5-7].

ChatGPT could potentially revolutionize the way of scholarly manuscript drafting. Nevertheless, there are several serious concerns that need to be addressed initially. First and foremost, the content it produces is prone to bias, as ChatGPT is not able to critically appraise its resources’ limitations or verify if they are biased or not. Moreover, its database is not up-to-date, as it is limited only up to September 2021. ChatGPT could prove to be unreliable, at least to some degree, since it can present inaccurate information as convincing and realistic [8]. These aspects are interestingly pointed out by the OpenAI team as well, deeming human oversight necessary.

It is assumed that it is really challenging, especially for reviewers, to identify whether any scientific content, such as an abstract, is produced exclusively by ChatGPT [2]. Taking also into consideration that ChatGPT can produce scientific abstracts with imaginary numerical data, it is apparent that this issue could pose a worrisome problem to the research community, as “forged” abstracts could potentially be presented at conferences or other scientific meetings.

Although it has been speculated, it has not yet been thoroughly examined, how difficult it is for a scientist, to differentiate between an AI-generated abstract to a human-written one. Therefore, it is up to future research to shed more light on these issues.

The purpose of this study is to assess the competence of reviewers to identify if an abstract is AI-generated or human-written. Expertise on the subject and the level of prior research experience are examined as possible confounders.

## Materials and methods

Seven blinded reviewers were asked to review 21 abstracts and identify which were AI-generated and which were written by humans. Six blinded reviewers were orthopaedic surgeons and were divided into two groups. The first group was formed by three orthopaedic residents with limited research experience (OR - Orthopaedic Residents), whereas the second group consisted of three professors of orthopaedics with extensive research background and experience as reviewers in peer-reviewed journals (OP - Orthopaedic Professors). The seventh reviewer was a non-orthopaedic doctor with extensive experience in research and a reviewer, who acted as a control in terms of orthopaedic-related expertise.

The leading author undertook the abstract selection. Ten abstracts, from high-impact-factor orthopaedic journals, were randomly chosen, while the type as well as topic of the studies varied [9-18]. For the formation of the 11 AI-generated abstracts, ChatGPT (May 24 Version) was utilized. The prompt used in order to produce the abstracts was “I want an accurate and scientific abstract regarding «topic of the study»” (Supplementary tables in the appendices)). The total of the 21 abstracts were only modified visually in terms of format, by removing the authors’ affiliations and any subtitle when present, but without modifying the content.

AI-generated abstracts were controlled by plagiarism detectors, and so did the original abstracts, mainly for evaluation purposes. Two of the most popularized AI detectors (ContentAtScale, GPTZero) were utilized, and their performance for the detection of AI-generated abstracts was evaluated as well.

A structured interview was conducted at the end of the survey in order to identify the decision-making process used by each reviewer. We asked the reviewers to identify which elements helped them to reach their decision. The structured interview of the reviewers focused on the following fields: Repetitive language, use of elaborate wording, general context, aim identification and relevance, statistical analysis/presentation of the results, and structure/applicability of the conclusion.

IBM SPSS Statistics for Windows, Version 23 (Released 2015; IBM Corp., Armonk, New York, United States) was utilized for the statistical analysis of the data. The sensitivity, the specificity, and the positive predictive values of the reviewers were calculated, and the statistical differences were checked with the Chi-squared test.

## Results

The OR group managed to identify correctly 34.9% of the abstracts’ authorship (19%, 28.5%, and 57.1%). The OP group identified correctly 31.7% (28.5%, 33.3%, and 33.3%). The non-orthopaedic control was identified correctly by 76.2% of the abstracts’ authors (Table 1).

**Figure 1:**
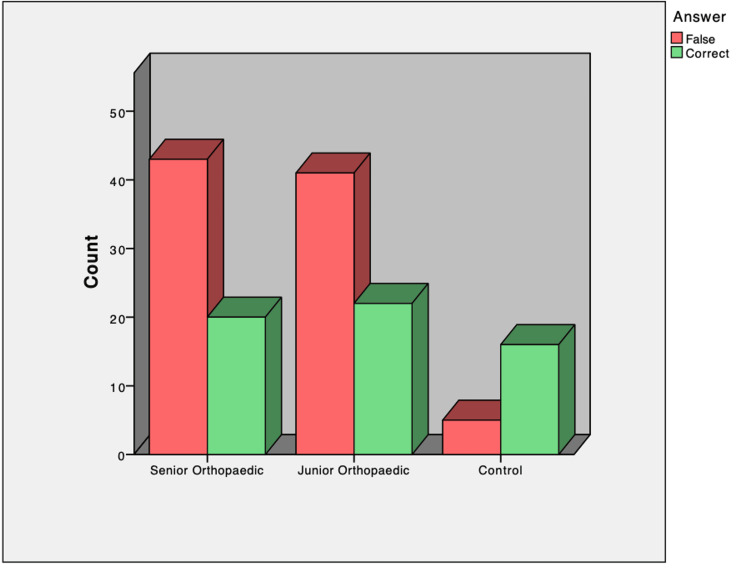
Graph showing the reviewers’ results in terms of the ability of authors’ identification

There was no statistically significant difference between the OR and OP groups (Chi-squared, p=0.85). There was a statistical difference between the answer of the control and OR group (Chi squared, p,0.002) and the control and OP group (chi-squared, p<0.005).

The sensitivity for AI identification was 10/33 (30.3%) for the OR group, 7/23 (30.4%) for the OP group, and 8/11 (72.7%) for the control. The specificity (ability to exclude AI abstracts) was 12/30 (20%) for the OR group, 13/30 (43,3%) for the OP group, and 8/10 (80%) for the control. The OR group had a positive predictive value for AI of 10/28 (35.7%), the OP group 7/24 (29.1%), and the control 8/10 (80%). The results are summarised in Table 1.

**Table 1:** Summary of the reviewers’ results

	Orthopaedic resident Group			Orthopaedic Professor Group			Non-Orthopaedic Control		
	False	Correct	Total	False	Correct	Total	False	Correct	Total
AI	23	10	33	26	7	33	3	8	11
Human	18	12	30	17	13	30	2	8	10
Total	41	22	63	43	20	63	5	16	21

All abstracts were checked for plagiarism by a plagiarism detection programme. All AI-generated abstracts were 100% unique (0% plagiarism). Plagiarism was identified in the human written abstracts with a mean percentage of 93.9% plagiarism (range 86-100%), as these abstracts were already published.

We subsequently utilised two different AI detectors (Contentatscale and GPTZero). The first AI detector (Contentatscale) gave inconclusive results for 7/21 abstracts (33.3%), of which 4 were AI generated and 3 written by humans. For the remaining 14 abstracts, this program correctly identified 9/14 (64.3%). For the conclusive answers, this AI program had sensitivity in detecting AI authorship 42.9% (3/7), specificity 85.7% (6/7), and positive predictive value 75% (3/4). In total, this AI detector managed to identify correctly only 9/21 (42.9%) of the abstracts’ authors.

The second AI Detector (GPTZero) gave an inconclusive answer for 6/21 abstracts (28.5%), from which 5 were AI generated and only one written by humans. It managed to identify correctly 14/15 (93.3%) of the abstracts’ authorship. For the conclusive answers this AI detector program had sensitivity 83.3% (5/6), specificity 100% (9/9), and positive predictive value 100% (5/5).

The results of this structured interview are summarised in Table 2. The OR group focused mainly on identifying language patterns and repetitions which were considered as AI-generated. The OP group concentrated mainly on the general context of the abstract and the applicability of the conclusion, aspects considered as human generated ones. On the other hand, the control was the only one evaluating the statistical aspect of the abstracts. This reviewer mainly focused on statistical presentation mistakes that were attributed to human writers. In contrast, when the statistical data appeared flawless, he believed the abstract was likely authored by AI.

**Table 2:** Structured interview results

	Orthopaedic Residents Group			Orthopaedic Professors Group			Non-Orthopaedic Control
	1^st^ Reviewer	2^nd^ Reviewer	3^rd^ Reviewer	4^th^ Reviewer	5^th^ Reviewer	6^th^ Reviewer	7^th^ Reviewer
Repetitive Language	X	X	X			Χ	X
Use of elaborate wording	X	X					
General context	X	X		X	Χ		
Aim identification and relevance		X		X			
Statistical analysis/presentation of the results							X
Structure/applicability of the conclusion	X		X	X	Χ	Χ	

As demonstrated previously, the control excelled in distinguishing between human and AI-generated content, outperforming the other two groups in identifying the authors of the text. Note that the non-orthopaedic control had special interest in statistics and no orthopaedic background. However, emphasizing on language patterns and repetitions, as well as assessing the general context of the abstract and the applicability of the conclusion, did not significantly contribute to achieving a high level of accuracy in determining the correct authorship of the abstracts

## Discussion

The most important finding of the current study is that neither the reviewers nor the AI detector programs were reliable in identifying correctly the authors of the abstracts included in the study. Additionally, neither expertise nor research background was shown to have any meaningful impact on the outcome. However, statistical proficiency, as well as diligent attention to detail with respect to statistical data presentation, may help to recognize a human-generated abstract. Nonetheless, it is difficult to identify the AI-written abstracts. As doctors from the relevant speciality focus more on the context and its applicability, it might be plausible that this obscures the identification of the AI output. Focus on non-medical elements, as the repetitive and predictable language might give hints for derivation from an AI programme. Furthermore, mistakes in language, statistical analysis presentation etc, could be produced by humans rather than computers. AI detection programs have high specificity (they can identify human authors correctly), but their sensitivity in detecting AI-generated abstracts varies significantly. The plagiarism tool was proven irrelevant, as AI-generated abstracts were 100% unique.

There is only one other study that also examines the ability of reviewers to identify an AI-written abstract. Although the methodology and design of this study differ, the conclusion stays the same; It is exceptionally hard to differentiate between AI- and human-written abstracts, as both human reviewers and AI detectors were not able to reliably detect an AI-generated abstract [19].

There are undisputedly certain benefits from the right use of ChatGPT and similar AI programmes. Mainly, its impressive linguistic features could be proven valuable in several healthcare fields. The production of general healthcare leaflets as well as other patient education material (post-operative instructions, rehabilitation protocols) could be simplified substantially. Furthermore, the content of these informative resources could easily be reformed by ChatGPT, in order to improve its readability and accessibility for individuals from diverse socioeconomic backgrounds, with various language proficiency levels [20]. Additionally, non-native English speakers could capitalize on ChatGPT’s linguistic skills, simplifying the manuscript drafting and possibly saving some expenses in the process, as Language services probably won’t be a necessity anymore.

Nevertheless, authors should absolutely not blindly rely on AI-generated texts. As already stated, ChatGPT’s content is not only prone to bias, but it can also include inaccurate and outdated information. Therefore, researchers should not expect scientific-safe content, and human oversight is without a doubt necessary. Regardless, ChatGPT can present this flawed data in a deceitful manner, and topped as well with imaginary statistics, to form eventually an abstract that could result in any conference, promoting false knowledge. Thus, more sophisticated AI detector tools must be developed in the near future to successfully reduce this possibility. In the meantime, reviewers must be alarmed, thoroughly scrutinize every abstract and probably demand more proofs (statistical analysis report, ethical board approval number), that also need to be validated by the Institution.

This study is not free of limitations. Although the reviewers were blinded, they did know that there were some AI-generated abstracts, hence introducing bias to a certain extent. However, as the main aim of the study was to assess the reviewer’s ability to differentiate between AI- and human-written abstracts, this drawback was unavoidable. The number of reviewers was relatively small, but it was still larger than the previous study. Additionally, we tried to compensate for that fact, by including controls for both, expertise and research experience. It is also important to acknowledge that while the study included a non-orthopaedic expert as a control for expertise, the representation of various specialties was limited. This limitation may have implications for the generalizability of the findings and warrants consideration in future research endeavours. Finally, we only included two of the most popular, widely available AI detectors. Although we believe that these results probably apply to most of the current publicly available AI detectors, it is possible that more sophisticated software has already been developed but has limited access.

## Conclusions

Abstracts written by ChatGPT can successfully deceive human reviewers, regardless of their previous research background and experience as reviewers. Development of advanced AI detectors is mandatory to prevent “false” abstracts from being presented at scientific conferences, as the presently available detectors are still underperforming. Focus on linguistic or statistical data presentation errors could help differentiate between AI- and human-written abstracts. Future research should further elucidate which elements could help reveal an AI-generated abstract.

## Appendices

**Table 3:** AI-generated abstracts

N.	Title	Abstract
1.	Prospective Observational Study of Radial Head Arthroplasty Outcomes: A Two-Year Follow-Up	Radial head fractures are a common injury, and radial head arthroplasty (RHA) is a well-established surgical treatment option. However, there is still limited evidence regarding the long-term outcomes of RHA. This prospective observational study aims to evaluate the outcomes of RHA in patients with radial head fractures and to identify any factors that may affect the outcomes. A total of 50 patients with acute or chronic radial head fractures underwent RHA using a modular metallic implant. The outcomes were evaluated using the Mayo Elbow Performance Score (MEPS), range of motion (ROM), and radiographic evaluation at 6 months, 1 year, and 2 years postoperatively. The mean MEPS improved from 56 preoperatively to 85 at 2 years postoperatively (p<0.001). The mean flexion-extension ROM improved from 114 degrees preoperatively to 132 degrees at 2 years postoperatively (p<0.001). The mean pronation-supination ROM improved from 124 degrees preoperatively to 147 degrees at 2 years postoperatively (p<0.001). There were no significant differences in the MEPS or ROM between patients with acute or chronic fractures. However, patients with associated injuries such as elbow dislocation had significantly lower MEPS at 2 years postoperatively (p<0.05).Radiographic evaluation showed that all implants were well-positioned, with no signs of loosening or implant-related complications at the 2-year follow-up. In conclusion, this prospective observational study demonstrates that RHA is a reliable treatment option for patients with acute or chronic radial head fractures, with significant improvements in functional outcomes and ROM at 2 years postoperatively. Associated injuries such as elbow dislocation may negatively impact the outcomes. Further long-term studies with larger sample sizes are needed to confirm these findings and to evaluate the durability and potential complications of RHA.
2.	Anterior Cruciate Ligament Reconstruction using the All-Inside Technique: A Systematic Review of Outcomes	Anterior cruciate ligament (ACL) reconstruction is a common surgical procedure used to treat ACL injuries. The all-inside technique is a newer approach to ACL reconstruction that utilizes smaller incisions and allows for a more anatomically accurate graft placement. This systematic review aims to summarize the current evidence on the outcomes of ACL reconstruction using the all-inside technique. A comprehensive search was conducted using PubMed, Embase, and Cochrane Library databases to identify studies that reported outcomes of ACL reconstruction using the all-inside technique. A total of 14 studies met the inclusion criteria, with a total of 1064 patients. Overall, the studies reported high rates of patient satisfaction, with 89.4% of patients reporting good or excellent outcomes at a mean follow-up of 36 months. The all-inside technique was also associated with low rates of complications, including infection and graft failure. Functional outcomes, including knee stability and range of motion, were also favorable, with the majority of patients returning to pre-injury activity levels. The studies showed that the all-inside technique provides good graft placement and preservation of the joint anatomy, which may contribute to improved functional outcomes.However, the studies did report a relatively high rate of meniscal injuries in patients who underwent ACL reconstruction using the all-inside technique, which may be due to the smaller incisions and increased difficulty in visualization during the procedure. In conclusion, ACL reconstruction using the all-inside technique is associated with high rates of patient satisfaction and low rates of complications. The technique provides good graft placement and preserves joint anatomy, which may contribute to improved functional outcomes. However, the potential for meniscal injuries should be considered when choosing this approach. Further research is needed to compare the all-inside technique to other approaches to ACL reconstruction.
3.	Partial Meniscectomy: A Review of Current Evidence and Outcomes	Partial meniscectomy is a common surgical procedure for the treatment of meniscal tears in the knee joint. Despite its widespread use, there is still debate regarding the effectiveness of this procedure in terms of pain relief and functional outcomes. This review aims to summarize the current evidence on the outcomes of partial meniscectomy and to identify any areas of controversy or uncertainty. Multiple studies have demonstrated that partial meniscectomy can provide significant pain relief and improvement in knee function in patients with meniscal tears. However, the degree of improvement varies based on several factors, including the location and extent of the tear, patient age and activity level, and pre-existing joint degeneration. One area of controversy is the long-term outcomes of partial meniscectomy. While many studies report good outcomes up to 10 years post-surgery, some research suggests that patients may experience a gradual decline in function and increased risk of osteoarthritis over time. Another area of uncertainty is the optimal timing for surgery. While early intervention may be beneficial for some patients, others may benefit from conservative management and physical therapy before considering surgical options. In conclusion, partial meniscectomy can provide significant pain relief and functional improvement in patients with meniscal tears. However, the long-term outcomes and optimal timing for surgery remain areas of debate and require further research. Clinicians should carefully consider individual patient factors and preferences when making treatment decisions for meniscal tears
4.	Ten-Year Survival Rate of Unicompartmental Knee Arthroplasty: A Systematic Review and Meta-Analysis of Clinical Studies	Unicompartmental knee arthroplasty (UKA) is a surgical technique that is commonly used to treat patients with isolated medial or lateral compartment osteoarthritis of the knee. This systematic review and meta-analysis aims to summarize the current evidence on the ten-year survival rate of UKA. A comprehensive search was conducted using PubMed, Embase, and Cochrane Library databases to identify clinical studies that reported on the ten-year survival rate of UKA. A total of 21 studies met the inclusion criteria, with a total of 5414 patients. The meta-analysis showed a ten-year survival rate of 90% (95% CI 87%-93%) for UKA. Subgroup analysis based on implant type showed no significant difference in survival rates between fixed-bearing and mobile-bearing implants (p=0.12). However, revision rates were significantly higher in patients with rheumatoid arthritis compared to those with osteoarthritis (OR 2.17, 95% CI 1.33-3.53, p=0.002). The studies also reported some factors associated with lower survival rates of UKA, including younger age, higher body mass index (BMI), and varus deformity. However, overall, UKA was found to have good long-term outcomes with high survival rates and low rates of complications. In conclusion, this systematic review and meta-analysis provides evidence to support the use of UKA as a viable treatment option for isolated medial or lateral compartment osteoarthritis of the knee, with a ten-year survival rate of 90%. While certain patient and implant factors may be associated with lower survival rates, careful patient selection and appropriate implant choice can lead to successful long-term outcomes. Further research is needed to compare the outcomes of UKA to total knee arthroplasty and to evaluate the long-term survival rates beyond ten years.
5.	Nailing versus Plate Fixation of Open Tibial Diaphyseal Fractures: A Systematic Review and Meta-Analysis of Outcomes	Open tibial diaphyseal fractures are common injuries that can be challenging to treat due to the risk of infection and nonunion. Nailing and plate fixation are two surgical techniques that are commonly used for the treatment of these fractures. This systematic review and meta-analysis aims to compare the outcomes of nailing versus plate fixation for open tibial diaphyseal fractures. A comprehensive search was conducted using PubMed, Embase, and Cochrane Library databases to identify studies that compared the outcomes of nailing and plate fixation for open tibial diaphyseal fractures. A total of 15 studies met the inclusion criteria, with a total of 1678 patients. The meta-analysis showed no significant difference in overall complication rates between nailing and plate fixation (OR 1.08, 95% CI 0.70-1.66, p=0.74). However, nailing was associated with a lower risk of deep infection (OR 0.47, 95% CI 0.31-0.70, p<0.001) and a shorter time to union (SMD -0.46, 95% CI -0.67 to -0.25, p<0.001) compared to plate fixation. Functional outcomes, including range of motion and return to work, were also comparable between the two techniques. However, nailing was associated with a shorter hospital stay and a lower risk of implant removal compared to plate fixation. The findings of this systematic review and meta-analysis suggest that while there is no significant difference in overall complication rates between nailing and plate fixation, nailing may offer some advantages in terms of lower risk of deep infection, shorter time to union, and shorter hospital stay. However, careful consideration of patient and fracture characteristics is necessary when making treatment decisions, and further research is needed to compare the long-term outcomes of these two techniques. In conclusion, both nailing and plate fixation are viable treatment options for open tibial diaphyseal fractures, with comparable functional outcomes. However, nailing may offer some advantages in terms of infection risk and time to union, and careful consideration of patient and fracture characteristics is necessary when making treatment decisions.
6.	Open Reduction and Internal Fixation of Clavicle Fractures: A Systematic Review of Outcomes	Clavicle fractures are a common injury, accounting for up to 5% of all fractures. Open reduction and internal fixation (ORIF) is a surgical technique that is commonly used to treat displaced or comminuted clavicle fractures. This systematic review aims to summarize the current evidence on the outcomes of ORIF for clavicle fractures. A comprehensive search was conducted using PubMed, Embase, and Cochrane Library databases to identify studies that reported outcomes of ORIF for clavicle fractures. A total of 31 studies met the inclusion criteria, with a total of 2151 patients. Overall, the studies reported good outcomes with ORIF for clavicle fractures, with high rates of fracture healing and low rates of complications. The majority of patients reported good functional outcomes, with return to work and sports activities within 3-6 months of surgery. However, the studies also reported some variability in outcomes based on fracture type and patient characteristics. For example, fractures with a high degree of comminution or displacement were associated with higher rates of complications, such as hardware failure or infection. Patients with a history of smoking or diabetes were also found to have a higher risk of complications. Despite these findings, the studies generally support the use of ORIF as a safe and effective treatment option for clavicle fractures. However, careful consideration should be given to patient characteristics and fracture type when deciding on the optimal treatment approach. In conclusion, ORIF is a common surgical technique for the treatment of clavicle fractures, with good outcomes reported in the literature. However, the optimal approach may depend on patient characteristics and fracture type, and careful consideration of potential complications is necessary when making treatment decisions. Further research is needed to compare the outcomes of ORIF to other treatment approaches for clavicle fractures.
7.	A Randomized Controlled Trial Comparing Reverse Total Shoulder Arthroplasty with Total Shoulder Arthroplasty in Proximal Humerus Fractures: Clinical and Radiographic Outcomes at 2 Years	Proximal humerus fractures are common injuries that can result in significant pain, loss of function, and disability. Total shoulder arthroplasty (TSA) has been used to treat severe proximal humerus fractures, but the use of reverse total shoulder arthroplasty (RTSA) is increasing due to potential advantages in stability and function. This randomized controlled trial (RCT) aims to compare the clinical and radiographic outcomes of RTSA and TSA in the treatment of proximal humerus fractures. A total of 80 patients with severe proximal humerus fractures were randomly assigned to undergo either RTSA or TSA. Clinical outcomes were evaluated using the Constant-Murley score (CMS) and the Disabilities of the Arm, Shoulder and Hand (DASH) score at baseline and at 6 months, 1 year, and 2 years postoperatively. Radiographic outcomes were evaluated using plain radiographs at the same time points. The results showed that both groups had significant improvements in CMS and DASH scores at all time points, with no significant difference between the two groups at 2 years postoperatively. However, the RTSA group had significantly better active forward flexion and external rotation at 1 year and 2 years compared to the TSA group (p<0.05). Radiographic outcomes showed no significant difference in implant position, loosening, or fracture between the two groups at 2 years. The RTSA group had a higher complication rate compared to the TSA group, with dislocation being the most common complication (10% vs. 2.5%, p=0.19). The revision rate was not significantly different between the two groups. In conclusion, this RCT found no significant difference in clinical or radiographic outcomes between RTSA and TSA in the treatment of proximal humerus fractures at 2 years. However, the RTSA group had significantly better active forward flexion and external rotation. While RTSA may provide better function, it is associated with a higher complication rate. Careful patient selection and appropriate surgical technique should be considered when deciding between RTSA and TSA for the treatment of proximal humerus fractures. Further research is needed to evaluate the long-term outcomes and complications of these procedures.
8.	A Randomized Controlled Trial Comparing Cementless Total Knee Arthroplasty with Cemented Total Knee Arthroplasty: Clinical and Radiographic Outcomes at 5 Years	Total knee arthroplasty (TKA) is a common surgical procedure used to treat patients with severe knee osteoarthritis. Cemented TKA has been the gold standard for many years, but cementless TKA is becoming increasingly popular due to the potential advantages of better fixation, reduced risk of cement-related complications, and improved long-term outcomes. This randomized controlled trial (RCT) aims to compare the clinical and radiographic outcomes of cementless TKA and cemented TKA at 5 years. A total of 150 patients with severe knee osteoarthritis were randomly assigned to undergo either cementless TKA or cemented TKA. Clinical outcomes were evaluated using the Knee Society Score (KSS) and the Western Ontario and McMaster Universities Arthritis Index (WOMAC) at baseline and at 1, 3, and 5 years postoperatively. Radiographic outcomes were evaluated using plain radiographs at the same time points. The results showed no significant difference in clinical outcomes between the two groups at any time point. The mean KSS and WOMAC scores improved significantly from baseline in both groups, with no significant difference between groups at any time point. Radiographic outcomes showed no significant difference in implant position, alignment, or loosening between the two groups at 5 years. However, the cementless group had a higher incidence of early postoperative pain (10% vs. 2%, p=0.04) and higher incidence of intraoperative fracture (8% vs. 0%, p=0.02) compared to the cemented group. There was no significant difference in the incidence of other complications or revision rates between the two groups. In conclusion, this RCT found no significant difference in clinical or radiographic outcomes between cementless TKA and cemented TKA at 5 years. However, the cementless group had a higher incidence of early postoperative pain and intraoperative fracture. Further research is needed to evaluate the long-term outcomes and complications of cementless TKA compared to cemented TKA.
9.	Association between Body Mass Index and Total Knee Arthroplasty Outcomes: A Cohort Study	Total knee arthroplasty (TKA) is a commonly performed surgical procedure for patients with end-stage knee osteoarthritis. Obesity is a known risk factor for knee osteoarthritis, but its impact on TKA outcomes is still controversial. This cohort study aims to evaluate the association between body mass index (BMI) and TKA outcomes. A total of 500 patients who underwent TKA were included in the study. The patients were categorized into three groups based on their BMI: normal weight (BMI <25), overweight (BMI 25-29.9), and obese (BMI ≥30). The primary outcome measures were the Knee Society Score (KSS) and the Western Ontario and McMaster Universities Osteoarthritis Index (WOMAC) score at 1 year post-surgery. Secondary outcome measures included perioperative complications and patient satisfaction. The results showed that the mean KSS and WOMAC scores improved significantly in all BMI groups at 1 year post-surgery (p<0.001). However, the improvement in KSS and WOMAC scores was significantly greater in the normal weight group compared to the overweight and obese groups (p<0.05). There was no significant difference in the improvement in KSS and WOMAC scores between the overweight and obese groups. The perioperative complication rates were similar among the BMI groups, with no significant difference in the rates of wound infection, deep vein thrombosis, or pulmonary embolism. However, the obese group had a significantly higher rate of superficial wound infection compared to the normal weight group (p=0.03). Patient satisfaction was high in all BMI groups, with no significant difference in satisfaction rates between the groups. In conclusion, this cohort study demonstrates that obesity is associated with inferior TKA outcomes in terms of functional improvement, as measured by KSS and WOMAC scores. However, the difference between the overweight and obese groups was not significant. Perioperative complication rates were similar among the BMI groups, but the obese group had a higher rate of superficial wound infection. These findings suggest that BMI may be an important factor to consider when evaluating TKA outcomes and counseling patients regarding their postoperative expectations
10.	Surgical Site Infections in Total Hip Arthroplasty: A Comprehensive Analysis of Risk Factors and Prevention Strategies	Surgical site infections (SSIs) following total hip arthroplasty (THA) pose significant challenges to both patients and healthcare providers. Understanding the risk factors associated with these infections is crucial for implementing effective preventive measures. This study aims to comprehensively analyze the risk factors for SSIs in THA procedures and evaluate strategies to mitigate infection rates. A systematic review of the literature was conducted, encompassing peer-reviewed articles, clinical guidelines, and meta-analyses published up to September 2021. Relevant databases including PubMed, Embase, and Cochrane Library were extensively searched using appropriate keywords. Studies reporting on risk factors and preventive strategies for SSIs in THA were included for analysis. Multiple risk factors contributing to SSI development in THA were identified. Patient-related factors such as advanced age, obesity, smoking, diabetes mellitus, and immunosuppression were consistently associated with an increased risk of infection. Surgical factors including prolonged operative time, intraoperative contamination, inadequate surgical technique, and improper wound closure techniques were also found to significantly influence SSI rates. In addition, hospital-related factors such as high patient turnover, inadequate infection control practices, and the presence of multidrug-resistant organisms were associated with increased infection risk. To mitigate the risk of SSIs, a multifaceted approach is essential. Preoperative optimization of modifiable risk factors, such as glycemic control in diabetic patients and smoking cessation programs, can significantly reduce infection rates. Strict adherence to aseptic techniques, appropriate antibiotic prophylaxis, and the use of laminar airflow systems in the operating room have shown promise in reducing the risk of contamination during surgery. Postoperative measures, including early detection of SSIs through surveillance programs, prompt wound assessment, and timely initiation of appropriate antimicrobial therapy, are crucial for successful management. This comprehensive analysis provides valuable insights into the multifactorial nature of SSIs following THA. By understanding and addressing the risk factors involved, orthopedic surgeons, infection control teams, and healthcare providers can collaboratively develop evidence-based preventive strategies. Further research is warranted to evaluate the effectiveness of these interventions and explore novel approaches to reduce the burden of SSIs in THA procedures.
11.	Comparison of the Efficacy and Safety of Nonsteroidal Anti-inflammatory Drugs versus Opioids for Pain Control in Patients Undergoing Total Knee Arthroplasty: A Retrospective Cohort Study	Nonsteroidal anti-inflammatory drugs (NSAIDs) and opioids are commonly used for pain control after total knee arthroplasty (TKA). However, there is still a lack of consensus on the optimal postoperative analgesic regimen. This retrospective cohort study aimed to compare the efficacy and safety of NSAIDs versus opioids for pain control in patients undergoing TKA. A total of 500 patients who underwent primary TKA were included in the study. Patients were categorized into two groups based on their postoperative analgesic regimen: NSAIDs group (n=250) and opioids group (n=250). The primary outcome measure was pain control, assessed using the visual analog scale (VAS) score at 24 hours, 48 hours, and 7 days postoperatively. The secondary outcome measure was the incidence of adverse events, including nausea, vomiting, constipation, and respiratory depression. The results showed that the NSAIDs group had significantly lower VAS scores compared to the opioids group at all three time points (p<0.05). The proportion of patients who achieved adequate pain control (VAS score ≤3) was significantly higher in the NSAIDs group compared to the opioids group at all three time points (p<0.05). In terms of adverse events, the incidence of nausea and vomiting was significantly lower in the NSAIDs group compared to the opioids group (p<0.05). However, there was no significant difference in the incidence of constipation or respiratory depression between the two groups. In subgroup analysis, patients with a history of chronic opioid use had significantly higher VAS scores and lower rates of achieving adequate pain control compared to patients without a history of chronic opioid use, regardless of the type of analgesic regimen used (p<0.05). In conclusion, this retrospective cohort study suggests that NSAIDs may be a more effective and safer option than opioids for pain control in patients undergoing TKA, with a lower incidence of adverse events such as nausea and vomiting. These findings suggest that NSAIDs may be a viable alternative to opioids in the management of postoperative pain in TKA patients, particularly in those with a history of chronic opioid use.

**Table 4:** Reviewers' questionnaire

	Question	Answer				
1.	How would you rate the use of repetitive language in the abstract?	1^Minimal^	2	3	4	5^Extensive^
2.	Did the abstract use elaborate wording? If so, how did it impact your evaluation?	1^Minimal^	2	3	4	5^Extensive^
3.	How well did the abstract provide a general context for the research?	1^Inadequate^	2	3	4	5^Clear^
4.	Was the aim of the research clearly identified and relevant to the field?	1^Inadequate^	2	3	4	5^Clear^
5.	How effective was the statistical analysis and presentation of the results?	1^Inadequate^	2	3	4	5^Very^
6.	Did the conclusion of the abstract have a clear structure and applicability to the research?	1^Inadequate^	2	3	4	5^Clear^
